# *Lactiplantibacillus plantarum* Ameliorated Morphological Damage and Barrier Dysfunction and Reduced Apoptosis and Ferroptosis in the Jejunum of Oxidatively Stressed Piglets

**DOI:** 10.3390/ani14223335

**Published:** 2024-11-20

**Authors:** Yu Liu, Junmeng Yuan, Wenshuo Xi, Zhisheng Wang, Huawei Liu, Kai Zhang, Jinshan Zhao, Yang Wang

**Affiliations:** College of Animal Science and Technology, Qingdao Agricultural University, Qingdao 266109, China; 20222203008@stu.qau.edu.cn (Y.L.); junmengyuan@stu.qau.edu.cn (J.Y.); xiwenshuo5213@163.com (W.X.); 17860820867@163.com (Z.W.); liuhuawei@qau.edu.cn (H.L.); zhangkai@qau.edu.cn (K.Z.); jszhaoqau@163.com (J.Z.)

**Keywords:** oxidative stress, piglets, ferroptosis, apoptosis, transcriptome

## Abstract

Oxidative stress induces apoptosis and ferroptosis, leading to intestinal injury of piglets. *Lactiplantibacillus plantarum* P8 (P8) has antioxidant capacity, but its roles in intestinal apoptosis and ferroptosis remain unclear. Here, 24 weaned piglets were assigned to three treatments: control (Con), diquat injection (DQ), and P8 supplementation + DQ injection (DQ + P8). The results showed that the increased jejunal oxidative stress, jejunal morphology impairment, and barrier dysfunction in the DQ-treated piglets were decreased by P8 supplementation. TUNEL and apoptosis-related gene expressions showed increased jejunal apoptosis of DQ-treated piglets; however, reduced apoptosis was observed in the DQ + P8 group. In addition, the mitochondrial morphology and ferroptosis-related gene expressions indicated elevated jejunal ferroptosis in the DQ-treated piglets, and the DQ + P8 treatment attenuated the ferroptosis. Transcriptome identified various differentially expressed genes (DEGs) between different treatments. KEGG analysis indicated that the DEGs were enriched in the PI3K-AKT, NF-κB, and apoptosis pathways. The expressions of key DEGs and key proteins in the PI3K-AKT and NF-κB pathways were further verified. In summary, our results indicate that P8 supplementation ameliorated jejunal oxidative stress, morphological damage, barrier dysfunction, apoptosis, and ferroptosis in the DQ-treated piglets. Moreover, the beneficial effect of P8 may be related to the regulation of PI3K/AKT and NF-κB pathways.

## Simple Summary

The gastrointestinal epithelium is important for maintaining intestinal homeostasis. During piglet production, excessive reactive oxygen species (ROS) can lead to intestinal mucosal oxidative injury, endangering the health of piglets. Moreover, oxidative stress has also been reported to be associated with apoptosis and ferroptosis. Probiotics are beneficial to intestinal health. In this study, piglets were fed a diet containing *Lactiplantibacillus plantarum* P8 (P8) for 29 days and injected with diquat (DQ) to induce oxidative stress on the 22nd day of the experiment in order to investigate the effects and mechanism of P8 on the intestinal oxidative injury. The results suggest that dietary supplementation of P8 contributed to an improvement in jejunal mucosal antioxidant capacity, microstructure and barrier function, and a decrease in apoptosis and ferroptosis. Jejunal transcriptome analysis further reveals that the beneficial effect of P8 may be related to the regulation of PI3K/AKT and NF-κB signaling pathways.

## 1. Introduction

The intestinal epithelial cells form a border in the intestinal tract, acting as a permeable barrier that allows the absorption of nutrients but inhibits the pathogens, bacteria, and toxins in the intestinal lumen from entering the circulation system [1]. Once the mucosal barrier is destroyed, intestinal structure damage, pathogens translocation, and inflammation progression will occur [2].

The intestinal epithelium is a dynamic tissue, and the turnover of the entire intestinal epithelium occurs every 3 to 5 days. The mature intestinal epithelium consists of rapidly proliferative cells in crypts from which cells differentiate into all kinds of mature intestinal epithelial cells. To maintain a consistent epithelial architecture, apoptosis serves as a mechanism to balance the rapid proliferation. Although apoptosis is essential for the maintenance of normal gut epithelial function, abnormal apoptosis is observed in the intestinal epithelial cells in a number of intestinal diseases. Ferroptosis, mediated by iron metabolism and lipid peroxidation, is a form of regulated cell death [3]. Ferroptotic cells have genetic, biochemical, and morphological characteristics, which are different from apoptotic, unregulated necrotic, and necroptotic cells [3]. Recent studies have also shown that ferroptosis is related to intestinal diseases, including inflammatory bowel disease, and colorectal cancer [4].

In intensive piglet production, various stressors, such as feed contamination, drug use, and diet ingredient changes, can result in the excessive production of reactive oxygen species (ROS) and induce intestinal oxidative injury [5]. Studies have shown that ROS and the resulting oxidative stress can induce apoptosis, while antioxidants block or delay apoptosis [6]. Jin et al. [7] suggested that diquat (DQ) treatment led to enhanced intracellular ROS production and apoptosis, and disrupted intestinal epithelial barrier function in intestinal porcine epithelial cells. Moreover, it is also reported that tissue injury caused by oxidative stress is closely associated with ferroptosis [8]. Xu et al. [5] found a higher level of ferroptosis in the jejunum and ileum of DQ-treated piglets.

Probiotics are well known to regulate intestinal health in humans and animals. *Lactiplantibacillus plantarum* P8 (P8) is a probiotic strain isolated from the natural fermented yogurt of the Inner Mongolian herder’s family. Our previous studies have revealed that supplementation with P8 could improve intestinal barrier function, antioxidant capacity, and inflammation in broilers [9,10,11]. However, there are few reports on the effects of P8 on the intestinal health of oxidatively stressed weaned piglets.

In this study, the weanling piglets were fed a basal diet with or without P8, followed by an intraperitoneal injection of DQ to trigger intestinal oxidative stress and injury [12,13]. This study aimed to explore whether P8 could improve intestinal health by regulating apoptosis and ferroptosis in the jejunal mucosa of weaned piglets using biochemical and transcriptome analyses.

## 2. Materials and Methods

### 2.1. Animal Ethical Approval

We conducted the trials in strict accordance with the guidelines of the Ethics and Animal Welfare Committee of Qingdao Agricultural University. This experiment was approved by the Ethics and Animal Welfare Committee of Qingdao Agricultural University (approval No: 20200813065).

### 2.2. Materials

P8 powder (1 × 10^11^ CFU/g) and DQ (Diquat dibromide monohydrate) were purchased from Beijing Scitop Bio-tech Co., Ltd. (Beijing, China) and Sigma-Aldrich Co., Ltd. (PS365; Burlington, MI, USA), respectively.

### 2.3. Experimental Design

Twenty-four 29-day-old weaned male Duroc × Landrace × Yorkshire piglets with similar initial body weight (7.50 ± 0.16 kg) were allocated to 3 treatments: Con, DQ, and DQ + P8, with eight piglets per pen. Piglets in the Con and DQ treatments were fed a basal diet, and piglets in the DQ + P8 treatments were fed a basal diet containing 1 × 10^8^ CFU/g P8. Moreover, on the 22nd day of the experiment, DQ (10 mg/kg body weight) was intraperitoneally injected into piglets on the DQ and DQ + P8 treatments [14]. The experimental period was 28 days. The basal diet was prepared based on NRC 2012 and the composition and nutrient levels are shown in Table 1 according to our previous study [13].

### 2.4. Sample Collection

On the 29th day of the experiment, all piglets were anesthetized with sodium pentobarbital (60 mg/kg BW), followed by carotid artery bleeding to cause death [15]. A portion of the dissected jejunum was fixed in 4% paraformaldehyde solution, and another portion (1 mm^3^) was fixed in the electron microscope fixation solution. Then, jejunal mucosal samples were scraped off and placed in liquid nitrogen immediately and then stored at −80 °C for further use.

### 2.5. Hematoxylin–Eosin (HE) Staining

The HE staining was performed according to our previous study [13]. The jejunal segments were fixed, embedded, hematoxylin-eosin (HE) stained, and then visualized with diaminobenzidine for 2 min. Perform the measurements of villus height and crypt depth according to the methods of Shan et al. [16] with an OLYMPUS microscope (OLYMPUS, Tokyo, Japan) and the HMIAS-2000 image analysis system (OLYMPUS, Tokyo, Japan).

### 2.6. Transmission Electron Microscope (TEM)

According to our previous study [13], the jejunal segments were fixed, dehydrated, cut, stained, and then captured using a transmission electron microscope (HITACHI HT7700 120 kv, Tokyo, Japan).

### 2.7. Biochemical Analysis

After the jejunal mucosa samples were homogenized [13], the activities of catalase (CAT, Cat. YJ22695), superoxide dismutase (SOD, Cat. YJ45986) and glutathione peroxidase (GSH-Px, Cat. YJ36985), and the concentrations of malondialdehyde (MDA, Cat. YJ54526) were determined in strict accordance with the instructions of the Enzyme-linked immunosorbent assay (ELISA) kits (Enzyme-Linked Biotechnology Co., Ltd., Shanghai, China). The absorbance was read by a microplate reader (SpectraMax iD3, Molecular Devices, Shanghai, China).

### 2.8. Real-Time Quantitative PCR (RT-qPCR)

Trizol reagent (Tiangen Biochemical Technology Co., Ltd., Beijing, China) was used to extract total RNA from jejunal mucosa. The TB Green^®^ Premix Ex TaqTM kit (Takara Biotechnology Co., Ltd., Dalian, China) was used to equip cDNA. RT-qPCR was then performed using a BioRad CFX96^TM^ Real-Time PCR system (Bio-Rad Laboratories, Hercules, CA, USA) [13]. *GAPDH* and *β-actin* were used as the reference genes. The forward and reverse primers of the genes (*ZO-1*, *Occludin*, *Claudin-1*, *Bax*, *Bad*, *Bcl-2*, *Caspase3*, *Caspase9*, *SLC7A11*, *GPX4*, *ACSL4*, *Nrf2*, *FSP1*, *SELENBP1*, *CYP4F3*, and *COL6A1*) are presented in Supplementary Table S1.

### 2.9. TUNEL

The apoptosis of jejunal tissues was assessed as described in the terminal dUTP-nick end labeling kit (Roche, Munich, Germany). Briefly, at 37 °C, jejunal sections were embedded with paraffin, then washed with PBS 3 times after 25 min in proteinase K working solution, washed with PBS 3 times after 20 min incubation in permeabilization solution, incubated with TUNEL reaction mixture for 1 h in a humidified chamber, and then filtered with 2-(4-amidinophenyl)-6-indolecarbamidine dihydrochloride (DAPI, Coolaber Science & Technology Co., Ltd., Beijing, China) for 10 min followed by 3 rinses with PBS. TUNEL-positive cells were observed by UV light microscopy (Nikon, Tokyo, Japan) as brilliant green.

### 2.10. Western Blotting

The total protein of the jejunal mucosa was extracted and quantified [13]. Protein samples were separated by electrophoresis and transferred to nitrocellulose membranes. After blocking the membranes for 30 min, the primary antibodies were incubated overnight (4 °C), and then rinsed and incubated with secondary antibodies for 60 min at room temperature. The antibodies, including β-actin (1:1000, Cat. GB11001), Claudin-1 (1:500, Cat. GB112543), Occludin (1:1000, Cat. GB11149), ZO-1 (1:500, Cat. GB111402), NF-κB p50 (1:800, Cat. GB115431), and AKT (1:2000, Cat. GB13427), were purchased from Servicebio Technology Co., Ltd. (Wuhan, China) and PI3K (1:2000, Cat. bsm-33219M), NF-κB p65 (1:2000, Cat. bs-0465R) and photoshop-IκBα (p-IκBα) (1:2000, Cat. bs-2513R) were obtained from Bioss Biotechnology Co., Ltd. (Beijing, China). An ECL detection system (Invitrogen iBright FL1000, Thermo Fisher Scientific, New York, NY, USA) was used to develop the blots. Image J software (Version 1.8.0.112, National Institutes of Health, Bethesda, MD, USA) was used to quantify relative protein expression [17].

### 2.11. Total RNA Extraction, mRNA Library Construction and Sequencing

Samples of jejunal mucosa (0.1 g) were sent to Panomix Biomedical Tech Co., Ltd. (Suzhou, China) for analysis using the NovaSeq 6000 platform (Illumina, San Diego, CA, USA). A 3 μg sample was taken to extract the mRNA, which was purified, fragmented, and generated [18]. End repair was performed by incubation with exonuclease/polymerase. The 3′ ends of the DNA fragments hybridized to Illumina PE adapter oligonucleotides. The AMPure XP system (Beckman Coulter, Beverly, CA, USA) was used to select the preferred cDNA fragments with a length of 400–500 bp. DNA fragments with ligated adaptor molecules at both ends of the 15-cycle PCR reaction were selectively enriched using the Illumina PCR Primer Cocktail. Products were purified (AMPure XP system) and quantified using the Agilent high-sensitivity DNA assay on a Bioanalyzer 2100 system (Agilent, Santa Clara, CA, USA).

The high-quality sequences used for further analysis were filtered by Cutadapt (v1.15) software, then mapped to the reference genome using HISAT2 v2.0.5 and statistically compared to the Read Count value for each gene using HTSeq (0.9.1), and the expression was normalized using FPKM. The screening condition is as follows: Expression difference multiple |log2FoldChange| > 1, *p* < 0.05 significantly. Bidirectional cluster analysis of all different genes and the enrichment analysis of the KEGG pathway of differential genes were performed using the R language Pheatmap (1.0.8) software package and ClusterProfiler (3.4.4) software, focusing on the significant enrichment pathway with *p*-value < 0.05. All raw gene data have been deposited in the NCBI (PRJNA1172817).

### 2.12. Statistical Analysis

All data were assessed by one-way ANOVA using SPSS 20.0. When effects were significant (*p* < 0.05), Tukey’s multiple range test was used to identify significant differences among the groups. GraphPad Prism 8 (GraphPad Software, La Jolla, CA, USA) was used to visualize the data by expressing the mean and standard error of the mean (SEM). The tabulated results are shown as means and SEM derived from the ANOVA error mean square. All means and comparison groups were considered statistically significant at *p* < 0.05.

## 3. Results

### 3.1. P8 Decreased the Jejunal Oxidative Stress of Piglets

**Figure 1:**
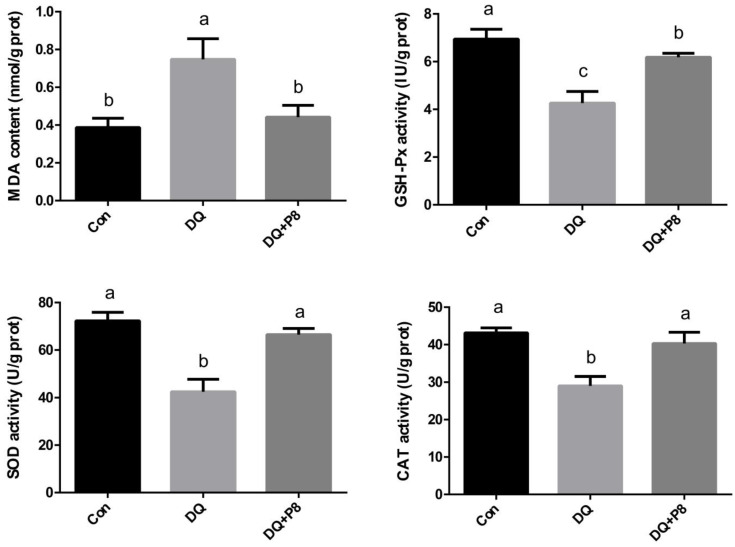
Effect of P8 on oxidative stress-related parameters in the jejunal mucosa of piglets. CAT, catalase; SOD, superoxide dismutase; MDA, malondialdehyde; GSH-Px, glutathione peroxidase. ^a,b,c^ Mean values within a row with no common superscript differ significantly (*p* < 0.05). *n* = 4.

According to Figure 1, the DQ treatment increased the MDA concentration (*p* < 0.05) and decreased the activities of SOD, CAT, and GPX (*p* < 0.05). On the contrary, the DQ + P8 treatment decreased the MDA concentration (*p* < 0.05) and elevated the activities of SOD, CAT, and GPX (*p* < 0.05) compared with the DQ group.

### 3.2. P8 Improved the Jejunal Morphology and Barrier Function of Piglets

**Figure 2:**
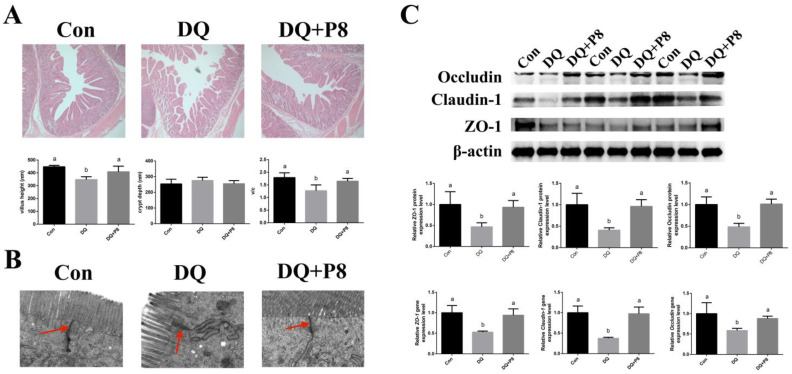
Effects of P8 on the jejunal morphology and barrier function of piglets. (**A**) HE staining sections (50×), *n* = 6. (**B**) TEM sections (15k×), *n* = 3. Red arrows indicate tight junctions. (**C**) Protein and gene expressions of tight junctions, *n* = 6. ^a,b^ Mean values within a row with no common superscript differ significantly (*p* < 0.05).

The HE staining results showed a decreased jejunal villus height and V/C ratio (*p* < 0.01) in the DQ-treated piglets. Moreover, the DQ + P8 treatment elevated the villus height and V/C ratio (*p* < 0.01) in comparison with the DQ treatment (Figure 2A). TEM results illustrated that the tight junctions were complete in the Con and DQ + P8 groups, but the DQ treatment caused unclear tight junctions and expanded paracellular spaces (Figure 2B). As for the tight junction-related gene and protein expressions, we found that the DQ treatment down-regulated the gene and protein expressions of *ZO-1*, *Claudin-1*, and *Occludin* (*p* < 0.01), while the DQ + P8 treatment up-regulated the gene and protein expressions of *ZO-1*, *Claudin-1*, and *Occludin* (*p* < 0.01) (Figure 2C, Figure S1).

### 3.3. P8 Decreased the Apoptosis in the Jejunal Mucosa of Piglets

**Figure 3:**
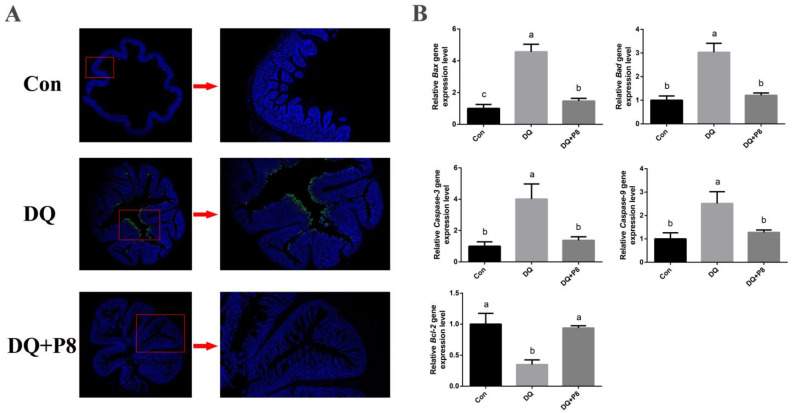
Effects of P8 on the apoptosis in jejunal mucosa of piglets. (**A**) TUNEL staining. The green spots represent apoptotic cells. *n* = 3. (**B**) Expressions of apoptosis-related genes. *n* = 6. ^a,b,c^ Mean values within a row with no common superscript differ significantly (*p* < 0.05).

TUNEL staining indicated a higher apoptosis level in the DQ treatment group, and the DQ + P8 treatment decreased the apoptosis level compared with the DQ treatment group (Figure 3A). Moreover, the DQ treatment also increased the gene expressions of *Bax*, *Bad*, *Caspase-3*, and *Caspase-9* (*p* < 0.01), and decreased the gene expression of *Bcl-2* (*p* < 0.01). Compared with the DQ treatment, the DQ + P8 treatment decreased the gene expressions of *Bax*, *Bad*, *Caspase-3*, and *Caspase-9* (*p* < 0.01), and increased the gene expression of *Bcl-2* (*p* < 0.01) (Figure 3B).

### 3.4. P8 Decreased the Ferroptosis in the Jejunal Mucosa of Piglets

**Figure 4:**
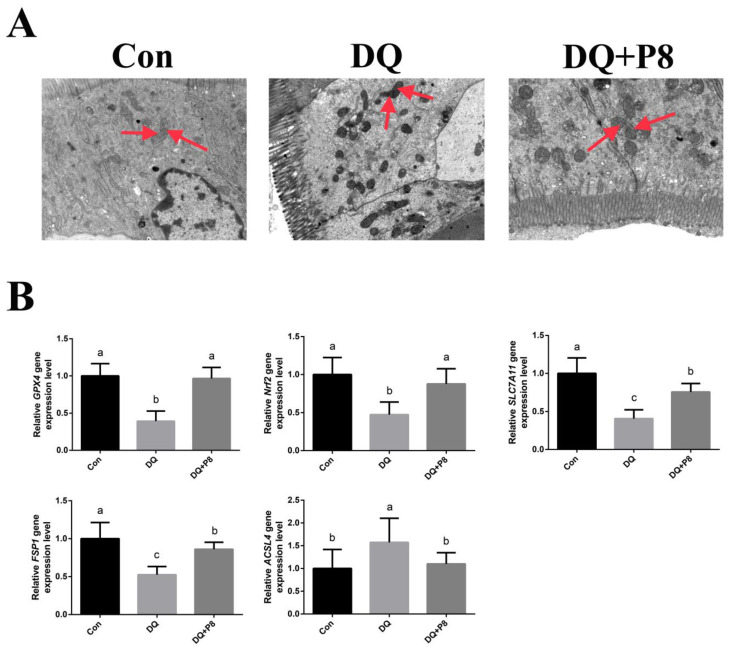
Effects of P8 on the ferroptosis in jejunal mucosa of piglets. (**A**) TEM sections, images were photographed under 5k× magnification, *n* = 3. Red arrows indicate mitochondria. (**B**) Effects of P8 on the ferroptosis genes in jejunal mucosa of piglets. *n* = 6. ^a,b,c^ Mean values within a row with no common superscript differ significantly (*p* < 0.05).

There were no obvious ferroptosis characteristics in the intestinal epithelial cells of the piglets in the Con group. However, ferroptosis characteristics were observed in piglets injected with DQ, including mitochondrial pyknosis and mitochondrial cristae reduction and dilatations. Interestingly, the P8 supplementation attenuated ferroptosis in the DQ-treated oxidatively stressed piglets (Figure 4A). Moreover, according to Table S1, the DQ treatment lowered the gene expressions of *FSP1*, *GPX4*, *SLC7A11*, and *Nrf2* (*p* < 0.01), and increased the gene expression of *ACSL4* (*p* < 0.01). In comparison with the DQ treatment, the DQ + P8 treatment increased the gene expressions of *FSP1*, *GPX4*, *SLC7A11*, and *Nrf2* (*p* < 0.01), and decreased the gene expression of *ACSL4* (*p* < 0.01) (Figure 4B).

### 3.5. Transcriptome

**Figure 5:**
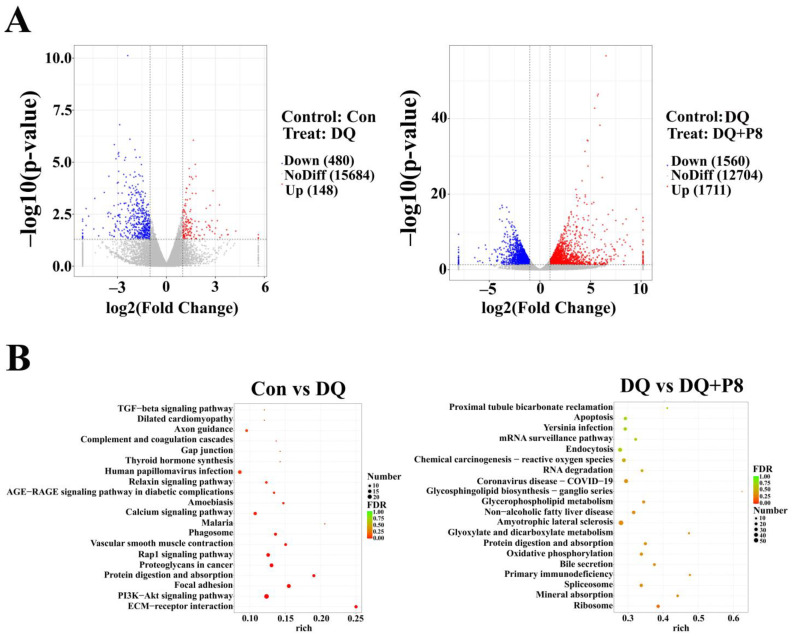
Effect of P8 on the transcriptome of the jejunal mucosa in piglets. (**A**) Volcano and (**B**) KEGG pathway in the jejunal mucosa of piglets. *n* = 3.

Compared with the Con group, 148 genes were up-regulated (*BTN1A1*, *MACROD2*, *CYP4F3*, etc.) and 480 genes were down-regulated (*FBLN2*, *SELENBP1*, *COL6A1*, etc.) in the DQ group (*p* < 0.05). In addition, 1711 genes were up-regulated (*TGFBR3L*, *SELENBP1*, *COL6A1*, etc.) and 1560 genes were down-regulated (*CYP4F3*, *RAB11FIP2*, etc.) in the DQ + P8 group (*p* < 0.05) compared with the DQ group (Figure 5A, Tables S2 and S3).

The functions of the DEGs were predicted by the KEGG analysis. The results demonstrated that the functions of DEGs between the DQ group and the Con group involved the PI3K/AKT signaling pathway, focal adhesion, and NF-κB. The functions of DEGs between the DQ group and the DQ + P8 group involved apoptosis, oxidative phosphorylation, NF-κB, etc. (Figure 5B, Table S4).

### 3.6. Validation of the Expressions of DEGs and Predicted Pathways

**Figure 6:**
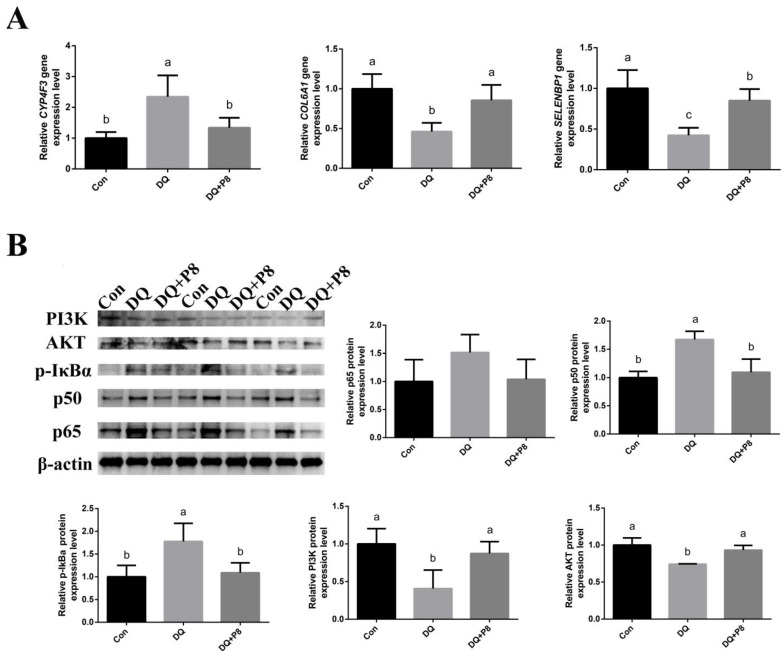
Validation of (**A**) DEGs, *n* = 6, and (**B**) predicted pathways, *n* = 3. ^a,b,c^ Mean values within a row with no common superscript differ significantly (*p* < 0.05).

We further validated the DEGs that related to apoptosis and ferroptosis by RT-qPCR. The results showed that the expression of *CYP4F3* was increased by the DQ treatments (*p* < 0.01), while the DQ + P8 treatment decreased *CYP4F3* expression (*p* < 0.01). On the contrary, the decreased expressions of *COL6A1* and *SELENBP1* in the DQ group (*p* < 0.01) were decreased by the DQ + P8 treatment (*p* < 0.01) (Figure 6A, Tables S2 and S3).

The predicted pathways that related to apoptosis and ferroptosis were also confirmed by Western blotting. Figure 6B indicated that the DQ injection activated the NF-κB signaling pathway by increasing the expressions of p-IκBα and p50 (*p* < 0.05), and DQ + P8 treatment decreased the expressions of p-IκBα and p50 (*p* < 0.05) in comparison with the DQ treatment. Moreover, the DQ treatment also inactivated the PI3K/AKT signaling pathway by decreasing the expressions of PI3K and AKT (*p* < 0.05), and the DQ + P8 treatment increased the expressions of PI3K and AKT (*p* < 0.05) compared with the DQ treatment (Figure S2).

## 4. Discussion

DQ is a bipyridyl herbicide, which has been commonly used as a model chemical for inducing intestinal oxidative damage in pigs [19,20]. In the present study, the DQ injection also increased the MDA level and decreased the activities of antioxidant enzymes. Moreover, damaged jejunal microstructure and barrier function were also observed in the DQ-treated piglets. Strain-specific probiotics have been reported to exert antioxidant capacity and improve the intestinal health of animals [21]. Here, decreased jejunal oxidative stress, as well as improved jejunal microstructure and barrier function, were observed in oxidatively stressed piglets receiving P8.

As apoptosis plays a crucial role in oxidative stress-induced gastrointestinal damage [22,23], we then explored enterocyte apoptosis. The TUNEL assay showed increased apoptosis in the jejunal mucosa of the DQ-treated piglets. Gene expressions also confirmed that the pro-apoptosis-related genes, such as *Bad*, *Bax*, *Capase-3*, and *Caspase-9*, were decreased, and the anti-apoptosis gene *Bcl-2* was increased by the DQ treatment. On the contrary, the dietary supplementation of P8 decreased the jejunal mucosal apoptosis manifested as the regulation of apoptosis genes and reduced TUNEL-positive cells. Similar to our results, publications also showed a decrease in apoptosis both in vitro and in vivo by the treatment of probiotics [24,25].

Ferroptosis, caused by the accumulation of iron-dependent lipid peroxides [3], is primarily influenced by iron metabolism and oxidative stress [26]. In addition, in recent years, researchers have found that there may be a relationship between apoptosis and ferroptosis. For instance, Lee et al. [27] showed that BAX-dependent mitochondrial pathway mediated the crosstalk between ferroptosis and apoptosis. Tang et al. [28] revealed that the Bcl-2 family proteins, the central regulators of apoptosis, are involved in abivertinib-induced ferroptosis. Therefore, we further examined the ferroptosis in the jejunal mucosa. Ferroptosis was primarily characterized by condensed mitochondrial membrane densities, diminished mitochondria crista, and ruptured outer membrane [29], which are different from other types of regulated cell death. Based on the TEM results, we found mitochondrial pyknosis, mitochondrial cristae reduction, and dilatations in piglets in the DQ group. However, no obvious mitochondria morphological damage was found in the DQ + P8 group. The ferroptosis-related gene expressions showed that the expression of *ACSL4* was increased, and the expressions of *FSP1*, *GPX4*, *SLC7A11*, and *Nrf2* were decreased in the DQ group. On the contrary, the expression of *ACSL4* was reduced, and the expressions of *FSP1*, *GPX4*, *SLC7A11*, and *Nrf2* were elevated in the DQ + P8 group. *ACSL4* contributes to the accumulation of lipid intermediates and plays a vital role in regulating ferroptosis sensitivity [30,31]. Increasing evidence suggests that ferroptosis can be induced by inhibiting cystine/glutamate transporter (SLC7A11/xCT) activity and down-regulating GPX4 and Nrf2 [32,33]. Moreover, FSP1 is a validated glutathione-independent ferroptosis suppressor [34]. Therefore, the changes in these ferroptosis-related genes and mitochondrial morphology imply the attenuation of ferroptosis in DQ-induced oxidatively stressed piglets. Previous studies also suggested decreased ferroptosis in animals treated with different probiotics [35,36]. Unfortunately, no other studies have reported data on intestinal ferroptosis in pigs treated with probiotics to serve for comparison with our results.

With the rapid development of next-generation sequencing, RNA sequencing has become an important tool to screen DEGs and study their functions. In the present study, the jejunal mucosal transcriptome demonstrated that the DEGs between the DQ and Con groups mainly included *CYP4F3*, *BTN1A1*, and *FBLN2*. The DEGs between the DQ and DQ + P8 included *SELENBP1*, *COL6A1*, and *CYP4F3.* Some of these DEGs were reported to be closely associated with ferroptosis and apoptosis. Zhao et al. [37] revealed that *SELENBP1* protects renal tubular epithelial cells from ferroptosis by up-regulating GPX4. Xu et al. [38] found that *CYP4F3* plays important roles in colorectal cancer development and is also considered a regulator of colorectal cancer cells to escape ferroptosis via Nrf2. Fan et al. [39] suggested that GPX4–PI3K/AKT–ferroptosis was a predominant pathway in deoxynivalenol-induced intestinal inflammation. They also examined the expressions of proteins (CDH17, CLDN4, COL6A1, and MYH9) that related to PI3K/AKT and tight junction pathways, and found that the expressions of CDH17 and COL6A1 were significantly decreased. Therefore, we verified the DEGs by RT-qPCR and found that the DQ treatment increased the expression of *CYP4F3* and decreased the expressions of *SELENBP1* and *COL6A1*, whereas the DQ + P8 treatment reversed these changes, which is in agreement with the DEGs results from the tanscriptome analysis.

The KEGG results of the jejunal mucosal transcriptome further showed that the DEGs between the DQ and Con groups were mostly enriched in the PI3K/AKT signaling pathway. As we mentioned, the DEG—*COL6A1* was associated with the tight junction and PI3K/AKT pathway, and the PI3K/AKT pathway can regulate the ferroptosis process [40]. Hence, we examined the expressions of the key proteins in the PI3K/AKT pathway by Western blotting. The results showed that the DQ treatment inactivated the PI3K/AKT pathway, and the DQ + P8 treatment activated the PI3K/AKT pathway by increasing the expression of PI3K and AKT. In addition, the NF-κB signaling pathway was also enriched both between the Con and DQ group and between the DQ and DQ + P8 group. Thus, the expression of related proteins, including p-IκBα, p50, and p65, was analyzed. We found that the expression of p-IκBα and p50 was up-regulated by the DQ treatment, and the DQ + P8 treatment inactivated the IκBα-p50 pathway. It is known that the NF-κB signaling pathway is involved in apoptosis [40,41]. Recent reports have also revealed the relationship between NF-κB and ferroptosis [42,43], and NF-κB may play a dual anti-ferroptosis and ferroptosis-promoting role, depending on the specific stimulus [43]. Zhao et al. [44] suggested that LPS triggered oxidative stress and ferroptosis in the liver through the NF-κB pathway. Furthermore, the functions of DEGs between the DQ group and the DQ + P8 group also involved apoptosis and oxidative phosphorylation, which were also in line with our finding that the elevated apoptosis level in the DQ treatment was decreased by the DQ + P8 treatment. In our previous study, by taking advantage of multi-omics, we found that dietary P8 supplementation improved growth and intestinal health by elevating the abundance of short-chain fatty acids (SCFAs)-producing bacteria, along with the changes of cecal content metabolites of broilers [11]. Moreover, Wang et al. also indicated that oral consumption of P8 elevated fecal SCFAs levels in adults [45]. It is reported that gut microbiota can regulate the NF-κB and PI3K/AKT signaling pathway by producing SCFAs [46,47]. Thus, we speculate that in the present study, the anti-apoptotic and anti-ferroptotic effects of P8 may be associated with the regulation of gut microbiota and SCFAs. In our future study, the roles of P8 on gut microbiota and SCFAs production will be explored to elaborate the detailed mechanisms.

## 5. Conclusions

In conclusion, our results indicate that the dietary P8 supplementation ameliorated jejunal oxidative stress, morphological damage, barrier dysfunction, apoptosis, and ferroptosis in DQ-treated piglets. Moreover, the beneficial effects of P8 may be related to the regulation of PI3K/AKT and NF-κB signaling pathways. It is suggested that P8 can be considered a highly efficient probiotic that improves the healthy farming of piglets.

## Figures and Tables

**Table 1:** Composition and nutrient levels of basal diets (%).

Item	Contents
Ingredients (%, as fed)	
Corn	60.00
Soybean meal	12.30
Extruded soybean	3.70
Fish meal	3.46
Whey powder	5.00
Soybean protein concentrate	6.60
Soybean oil	2.60
Sucrose	3.00
Limestone	0.60
CaHPO_4_	0.90
NaCl	0.10
*L*-Lys·HCl	0.50
*DL*-Met	0.10
*L*-Thr	0.10
*L*-Trp	0.04
Chloride choline	0.10
Premix ^1^	0.50
Acidifier	0.40
Total	100.00
Nutrient levels ^2^	
Digestible Energy, MJ/kg	14.73
Crude protein, %	19.03
Calcium, %	0.79
Available P, %	0.37
Digestible-lysine, %	1.33
Digestible-methionine, %	0.38
Threonine, %	0.74
Tryptophan, %	0.23

^1^ Premix provides: vitamin A 2000 IU; vitamin B1 2.0 mg; vitamin B2 4.0 mg; vitamin B6 5.0 mg; vitamin B12 0.02 mg; vitamin D3300 IU; vitamin E 20 IU; vitamin K3 3.0 mg; biotin 0.1 mg; folic acid 0.5 mg; pantothenic acid 15 mg; niacin 0.75 mg; copper (copper sulfate) 25 mg; iron (ferrous sulfate) 100 mg; manganese (manganese sulfate) 10 mg; zinc (zinc sulfate) 100 mg; iodine (potassium iodide) 0.30 mg; selenium (sodium selenite) 0.30 mg; Enzyme-linked 100 IU. ^2^ calculated values of nutrient levels.

## Data Availability

The data supporting this article have been included as part of the Supplementary Information.

## Supplementary Materials

The following supporting information can be downloaded at: https://www.mdpi.com/article/10.3390/ani14223335/s1. Table S1: Gene primers; Table S2: DEGs between the DQ and Con groups (top 10) and DEGs associated with apoptosis and ferroptosis; Table S3: DEGs between the DQ + P8 and DQ groups (top 10) and DEGs associated with apoptosis and ferroptosis; Table S4: NF-κB is a common predicted pathway in Con vs. DQ and DQ vs. DQ + P8; Figure S1: Western blots raw data of tight junction-related protein; Figure S2: Western blots raw data of PI3K/AKT and NF-κB signaling pathways.
