# Supplementary material for: Lactiplantibacillus plantarum Ameliorated Morphological Damage and Barrier Dysfunction and Reduced Apoptosis and Ferroptosis in the Jejunum of Oxidatively Stressed Piglets
Source: Animals (Basel). 2024 Nov 20;14(22):3335. doi: 10.3390/ani14223335 (PMC11591186; doi:10.3390/ani14223335)

Supplementary materials

Supplementary Table S1. Gene primers

Gene	Forward Primer sequence（5'-3')	Reverse Primer sequence（5'-3')
β-actin	CCCAAAGCCAACCGTGAGAA	CCACGTACATGGCTGGGGTG
GAPDH	CAGCAATGCCTCCTGTACCA	CCACGATGCCGAAGTTGTC
ZO-1	CGGCGAAGGTAATTCAGTGT	TCTTCTCGGTTTGGTGGTCT
Occludin	ATGCTTTCTCAGCCAGCGTA	AAGGTTCCATAGCCTCGGTC
Claudin-1	AGATTTACTCCTACGCTGGTGAC	GCAAAGTGGTGTTCAGATTCAG
Bax	GAGCAGATCATGAAGACAGGGG	AAGTAGAAAAGCGCGACCAC
Bad	CCGAGGAGGATGAAGGGACTGAG	AGGAACCCTGGAACTCGTCACTC
Bcl-2	CGGCGATGACTTCTCTCGT	TTGACGCTCTCCACACACAT
Caspase3	TGTGGGATTGAGACGGACAG	CGCTGCACAAAGTGACTGGA
Caspase9	GGCCAGTGGACATTGGTTCT	GGCCTTGGCAGTCAGGTT
SLC7A11	CGTCCTTTCAAGGTGCCGA	TAAAGCGAAAGGGCGACCAC
GPX4	TTACGGATTCTGGCCTTCCC	CCGTTCTTATCAATGAGGAACTTGG
ACSL4	GCCACCACTGCAACAAAACA	CAGGACCAGCAGAAGCTGAA
Nrf2	GCTACGGGATTGGGGTTTGG	AGACCTCAGTTCCCCCAAGA
FSP1	CCGGCGACAACCGGAG	TCCTTCATGTCCACCAGCAC
SELENBP1	TCATAACACACGCTCTCCCA	CAGGGGTCAAGGTTTCAGGG
CYP4F3	CCTGGTAGCTTCCTCTAGGTCA	TCCCACACAACTTCTTTCCCC
COL6A1	GATGGGGGAAAGGGGTGAAG	ACGTCGTTGTTGTCCTCTCC

Supplementary Table S2. DEGs between the DQ and Con groups (top 10) and DEGs associated with apoptosis and ferroptosis

RNA	Con (%)	DQ (%)	up/down	Log_2FC	*p*-value
BTN1A1	1.92	94.89	up	5.63	0.045
MACROD2	0.32	6.01	up	4.25	0.021
FBLN2	9.76	0.32	down	-4.92	0.011
ADH1C	149.74	4.97	down	-4.91	0.002
CPXM1	8.75	0.32	down	-4.76	0.004
CTNND2	7.03	0.32	down	-4.44	0.016
COL21A1	13.51	0.65	down	-4.38	0.001
ADAMTSL3	5.71	0.32	down	-4.14	0.035
CHRDL1	6.63	0.45	down	-3.87	0.015
PLXDC1	40.54	2.91	down	-3.80	0.000
SELENBP1	159.03	75.22	down	-1.08	0.004
COL6A1	783.39	250.94	down	-1.64	0.008
CYP4F3	5.00	48.54	up	3.28	0.042

Supplementary Table S3. DEGs between the DQ+P8 and DQ groups (top 10) and DEGs associated with apoptosis and ferroptosis

RNA	Con (%)	DQ (%)	up/down	Log_2FC	*p*-value
TGFBR3L	0.18	50.73	up	8.12	0.000
RAB11FIP2	114.97	0.44	down	-8.03	0.000
GNRH1	0.18	33.08	up	7.51	0.000
KCNRG	8.06	1464.04	up	7.51	0.000
SERPIND1	0.18	29.50	up	7.34	0.000
NUCB1	0.37	52.32	up	7.15	0.000
CPXM1	0.18	23.12	up	6.97	0.002
IFN-DELTA-8	0.98	114.23	up	6.87	0.000
CDC42SE2	2.98	323.69	up	6.76	0.000
EFCAB12	0.18	18.74	up	6.67	0.015
SELENBP1	42.24	119.67	up	1.50	0.027
COL6A1	142.18	474.39	up	1.74	0.026
CYP4F3	27.18	2.36	down	-3.52	0.037

Supplementary Table S4. NF-κB is a common predicted pathway in Con vs DQ and DQ vs DQ+P8

PathwayID	Pathway	Group	DEG number	*p*-value	FDR
Ssc04064	NF-kappa B signaling pathway	Con vs DQ	3	0.49	0.74
		DQ vs DQ+P8	15	0.43	1.00

Supplementary Figure S1. Western blots raw date of tight junction-related protein

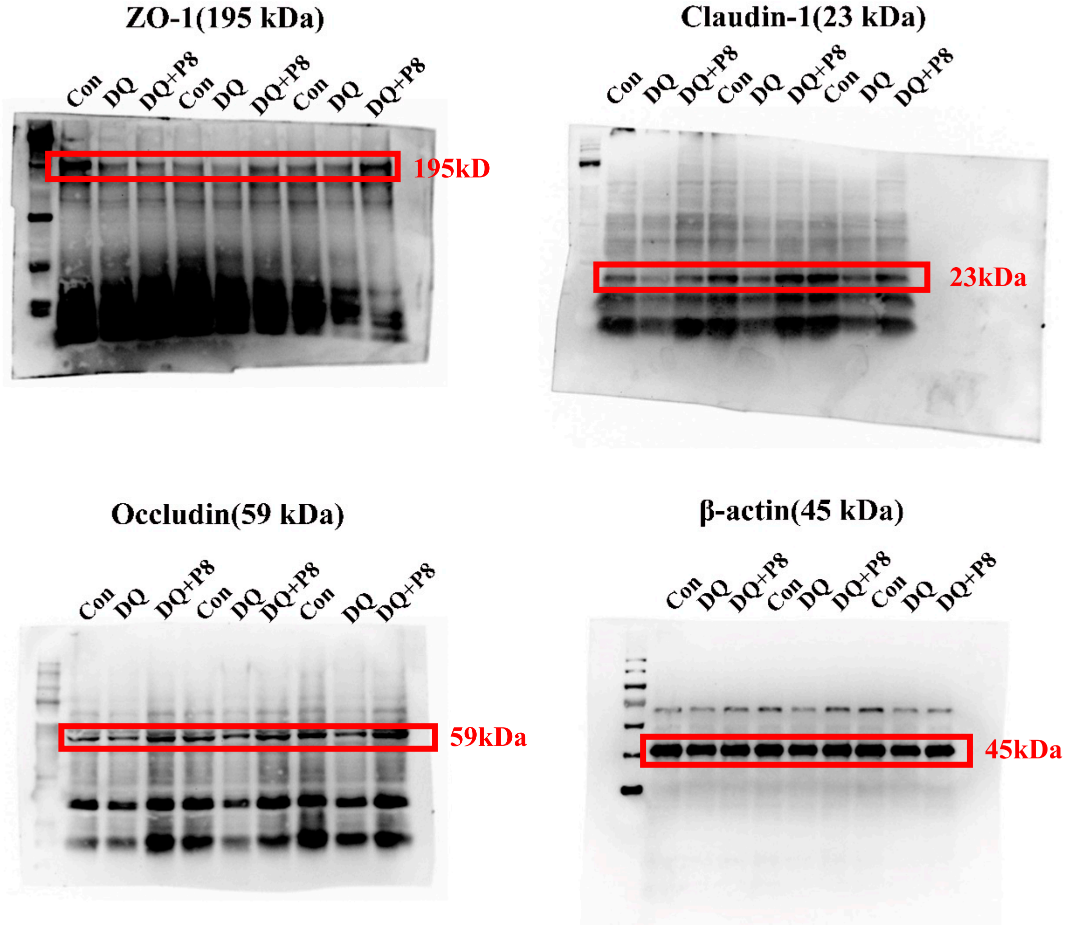

Supplementary Figure S2. Western blots raw date of PI3K/AKT and NF-κB signaling pathways

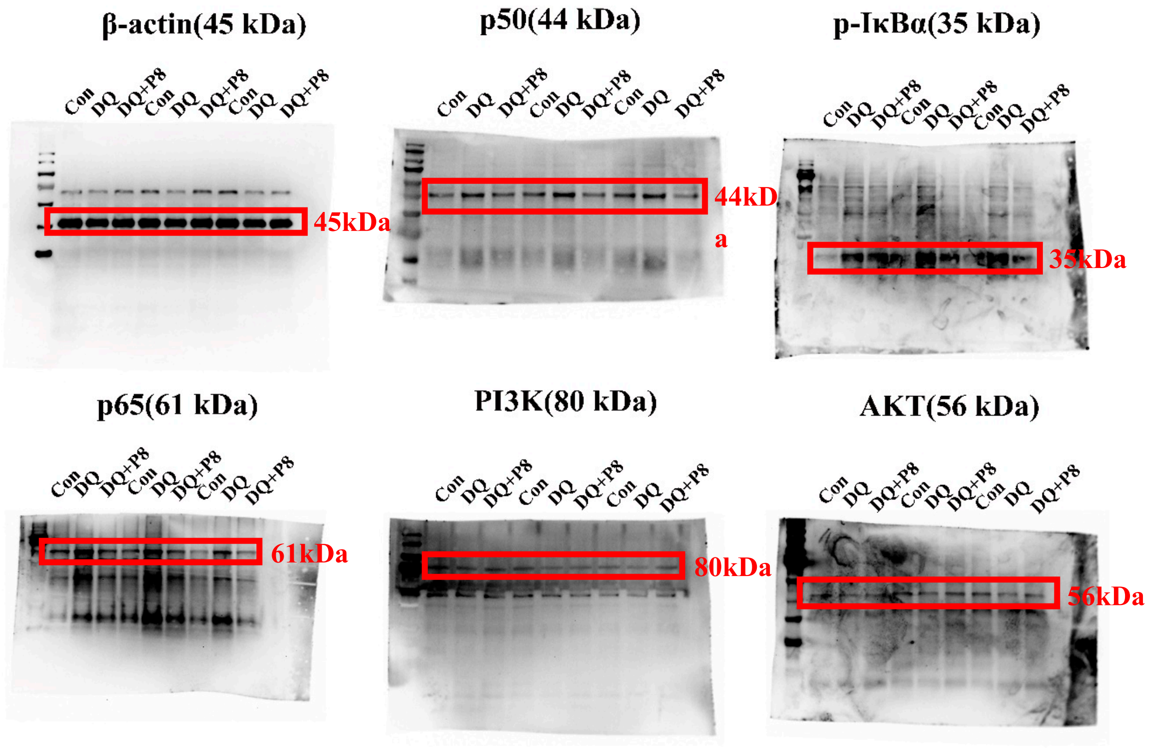

Supplement: Supplementary file 1 [file animals-14-03335-s001.zip › animals-3296882-supplementary.pdf]
